# Low-dose CBCT imaging of alveolar buccal bone adjacent to mandibular anterior teeth— a pilot study

**DOI:** 10.1007/s00784-022-04389-x

**Published:** 2022-02-01

**Authors:** Maurice Ruetters, Holger Gehrig, Dorothea Kronsteiner, Sara Doll, Ti-Sun Kim, Christopher J. Lux, Sinan Sen

**Affiliations:** 1 Section of Periodontology, Department of Operative Dentistry, University Hospital Heidelberg, Im Neuenheimer Feld 400, 69120 Heidelberg, Germany; 2 Department of Orthodontics, University Hospital Heidelberg, Im Neuenheimer Feld 400, 69120 Heidelberg, Germany; 3 Institute of Medical Biometry, University Hospital Heidelberg, Im Neuenheimer Feld 130.3, 69120 Heidelberg, Germany; 4 Department of Anatomy and Cell Biology, Heidelberg University, Im Neuenheimer Feld 307, 69120 Heidelberg, Germany

**Keywords:** Periodontitis, Radiology, Periodontal surgery, Orthodontics

## Abstract

**Objectives:**

Accurate description of buccal bone adjacent to mandibular anterior teeth is helpful for planning and monitoring periodontal and orthodontic treatment. Low-dose cone beam computed tomography (LD-CBCT) imaging has shown promising results for very small dental structures in animals. This study asserts that LD-CBCT is sufficiently accurate to measure buccal alveolar bone adjacent to human mandibular anterior teeth.

**Materials and methods:**

Buccal bone level adjacent to 16 mandibular anterior teeth from four human cadavers was measured radiographically using one high-dose (HD) CBCT protocol and two LD-CBCT protocols. The resulting radiographic measurements of buccal bone height (bl) and thickness (bt) were compared with reference probe and reflected-light microscopy measurements. Measurement medians and Bland–Altman plots were calculated, and a linear mixed model was used to compare raters and imaging modalities.

**Results:**

All regression coefficients were approximately 0, indicating high interrater, intrarater, and intermodality agreement. No significant differences were found between reference measurements and CBCT protocols. The mean differences for bl measurements were 0.07 mm (rater 1 [r1]) and 0.12 mm (rater 2 [r2]) for HD-CBCT; 0.07 mm (r1) and 0.13 mm (r2) for LD-CBCT-1; and 0.02 mm (r1) and 0.01 mm (r2) for LD-CBCT-2. For bt measurements, mean differences were 0.02 mm (r1) and 0.02 mm (r2) for HD-CBCT; 0.01 mm (r1) and 0.01 mm (r2) for LD-CBCT-1; and 0.00 mm (r1) and 0.01 mm (r2) for LD-CBCT-2.

**Conclusions:**

Within the limitations of the present study, LD-CBCT seems to be a precise method for describing buccal bone and its thickness adjacent to mandibular anterior teeth in this experimental setting.

**Clinical relevance:**

For the first time, this study showed LD-CBCT produces excellent results and is a reliable modality for imaging buccal bone in vitro. If clinical studies confirm these results, LD-CBCT could enable better treatment planning and monitoring at a radiation dose that is far lower than that of conventional HD-CBCT but similar to that of panoramic views.

**Supplementary Information:**

The online version contains supplementary material available at 10.1007/s00784-022-04389-x.

## Introduction

The buccal bone adjacent to the mandibular anterior teeth is a highly sensitive periodontal structure. It is characterized above all by its usually very delicate and thin anatomy [1, 2]. Its morphology influences both the periodontal and the peri-implant phenotype, which now play an important role in diagnosis and treatment planning [3–6]. Precise knowledge of the individual anatomy of the bone can be of great value to the periodontist, enabling them to precisely plan periodontal regenerative surgery, for example. It might also shorten the duration of surgery and possibly even allow more precise incision. This has already been demonstrated for furcation defects in molars [7]. Moreover, knowledge of the buccal bone is also of utmost interest to orthodontists. Detailed knowledge of the nature of the buccal lamella in terms of bone thickness and the presence of fenestrations might enable dentists to avoid undesirable treatment-induced damage, such as gingival recession resulting from overextended vestibular tooth movement [8].

In recent years, there have been new developments in cone beam computed tomography (CBCT) devices. Previously, the benefits of imaging in three dimensions were accompanied by a significantly higher radiation dose for the patient compared with standard two-dimensional procedures such as panoramic views [9–11]. New-generation machines offer “low-dose cone beam computed tomography” (LD-CBCT) protocols in addition to already established protocols. These protocols enable three-dimensional imaging of the maxillofacial region at a relatively low radiation dose, as little as 12–29 mSv [9]. In addition, as a result of further developments in high-resolution sensors, CBCT machines can now produce images down to a spatial resolution of 0.08 mm [12, 13]. However, it is unknown whether these devices can image the buccal bone of mandibular anterior teeth with sufficient contrast and sharpness if LD-CBCT protocols are used. A study of the suitability of LD-CBCT for imaging the vestibular lamella in pig jaws showed promising results [14]; however, because of differences between human and pig anatomy, the results of that study are not fully applicable to humans. The as low as diagnostically acceptable (ALADA) principle means that LD-CBCT is likely to be of interest for other indications requiring imaging of the buccal bone of mandibular anterior teeth, in addition to the ones mentioned above. For example, LD-CBCT could be used to monitor whether, and to what extent, bony regeneration occurs after recession coverage. Until now, this could only be determined by means of histological examinations and nonionizing ultrasound, which are not routinely available in dental practice, or by using conventional CBCT, but at a significantly higher radiation dose [9, 15–19].

The present study therefore aims to investigate whether LD-CBCT can sufficiently visualize the buccal osseous lamella adjacent to human mandibular anterior teeth.

## Materials and methods

This *ex vivo* study investigated 16 mandibular anterior teeth from four human hemisected cadaveric heads. The heads were from bodies donated to the Institute of Anatomy and Cell Biology of the University of Heidelberg and were preserved with 99% ethanol, glycerin, and 37% formalin. To ensure clear reproducibility of the image planes in the different acquisition modes, two depressions were made on the vestibular surface of the crown of each tooth by means of a round diamond burr (801L 314 016, Komet Dental, Gebr. Brasseler GmbH & Co. KG, Lemgo, Germany). All available mandibular anterior teeth (front teeth and canines) with an existing crown were included in the study. Restorations or carious lesions on the 16 teeth investigated or on other teeth did not constitute exclusion criteria. Implants were excluded.

### Cone beam computed tomography

**Fig. 1:**
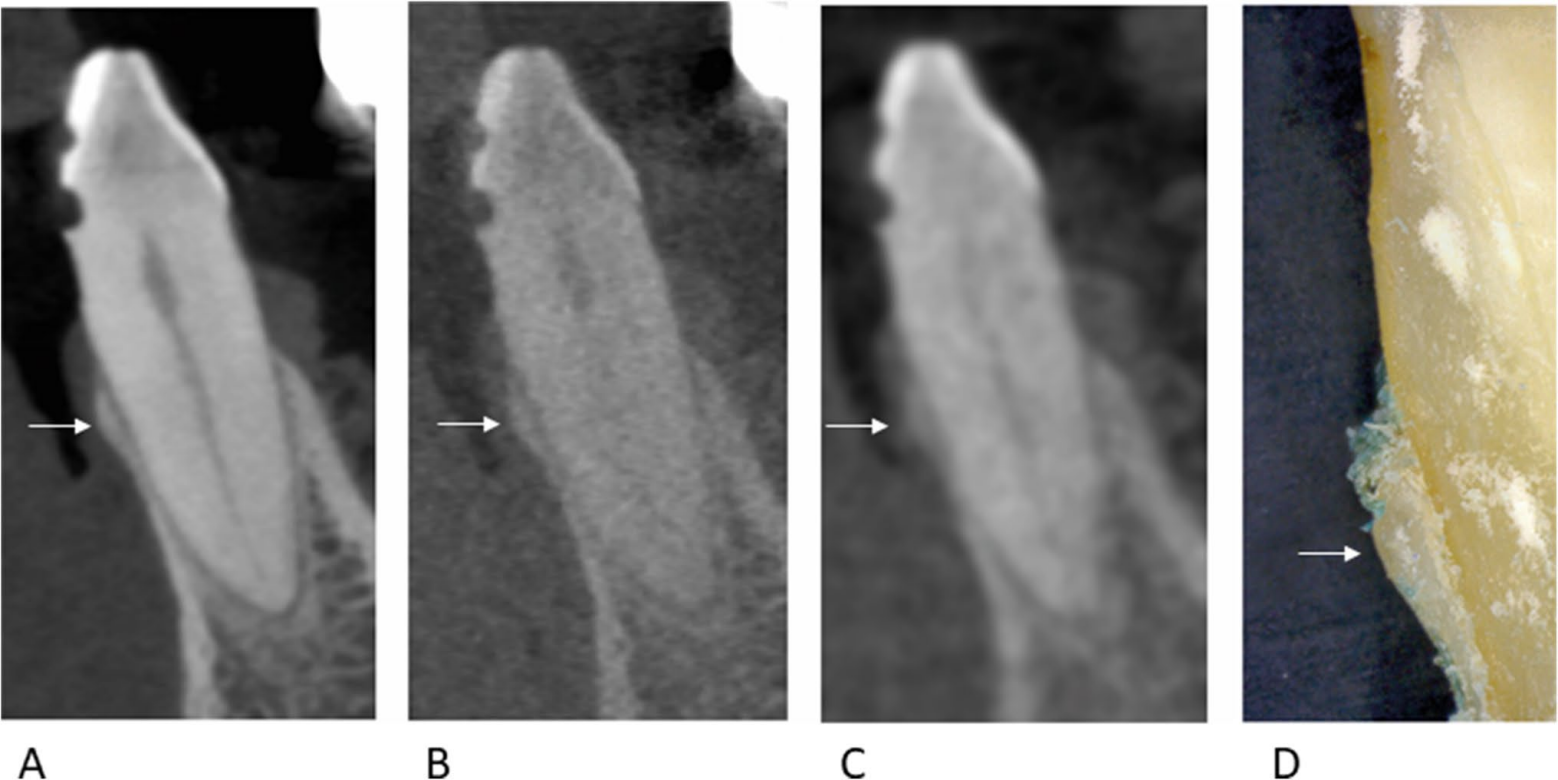
Sagittal planes of the three different CBCT protocols and reflected-light microscopy of tooth 32. **A** HD-CBCT. **B** LD-CBCT protocol 1. **C** LD-CBCT protocol 2. **D** Reflected-light microscopy. White arrows indicate the buccal lamina. CBCT, cone beam computed tomography. HD, high dose. LD, low dose

At the time of the radiographic investigations, the hemisected cadaveric heads, including the mandibles, were fully covered by soft tissue and by the adjacent muscles of the cheek. The tongue, neck muscles, base of the skull, and cervical vertebrae were also still present. The teeth were radiographically imaged using three CBCT protocols (Fig. 1): two LD-CBCT protocols of two different devices (Veraview X800, J. Morita Europe, Dietzenbach, Germany, and Orthophos 3D SL®, Dentsply Sirona, Bensheim, Germany) and a high-dose (HD) CBCT protocol of one device (Veraview X800).

The volumetric acquisition protocols were as follows:

- HD-CBCT protocol (Veraview X800): 17.9 s radiation time, 5 mA, 102 kV, field of view (FOV) 8 × 8 cm^2^, isotropic voxel size 0.125 mm, and dose area product (DAP) 1396.95 mGy cm^2^
- LD-CBCT protocol 1 (Veraview X800): 9.4 s radiation time, 1.6 mA, 72 kV, FOV 8 × 8 cm^2^, isotropic voxel size 0.125 mm, and DAP 87.19 mGy cm^2^
- LD-CBCT protocol 2 (Orthophos 3D SL): 2.1 s radiation time, 10 mA, 85 kV, FOV 8 × 8 cm^2^, isotropic voxel size 0.16 mm, and DAP 69 mGy cm^2^

During imaging, gel pads were used to imitate the other half of the head to achieve tissue-equivalent volumes and ensure the most lifelike absorption of radiation [20].

The heads were fixed in position by placing the throat in a tube, and they were oriented in accordance with the orientation lines specified by the manufacturer.

### Probe measurements and reflected-light microscopy — reference standards

**Fig. 2:**
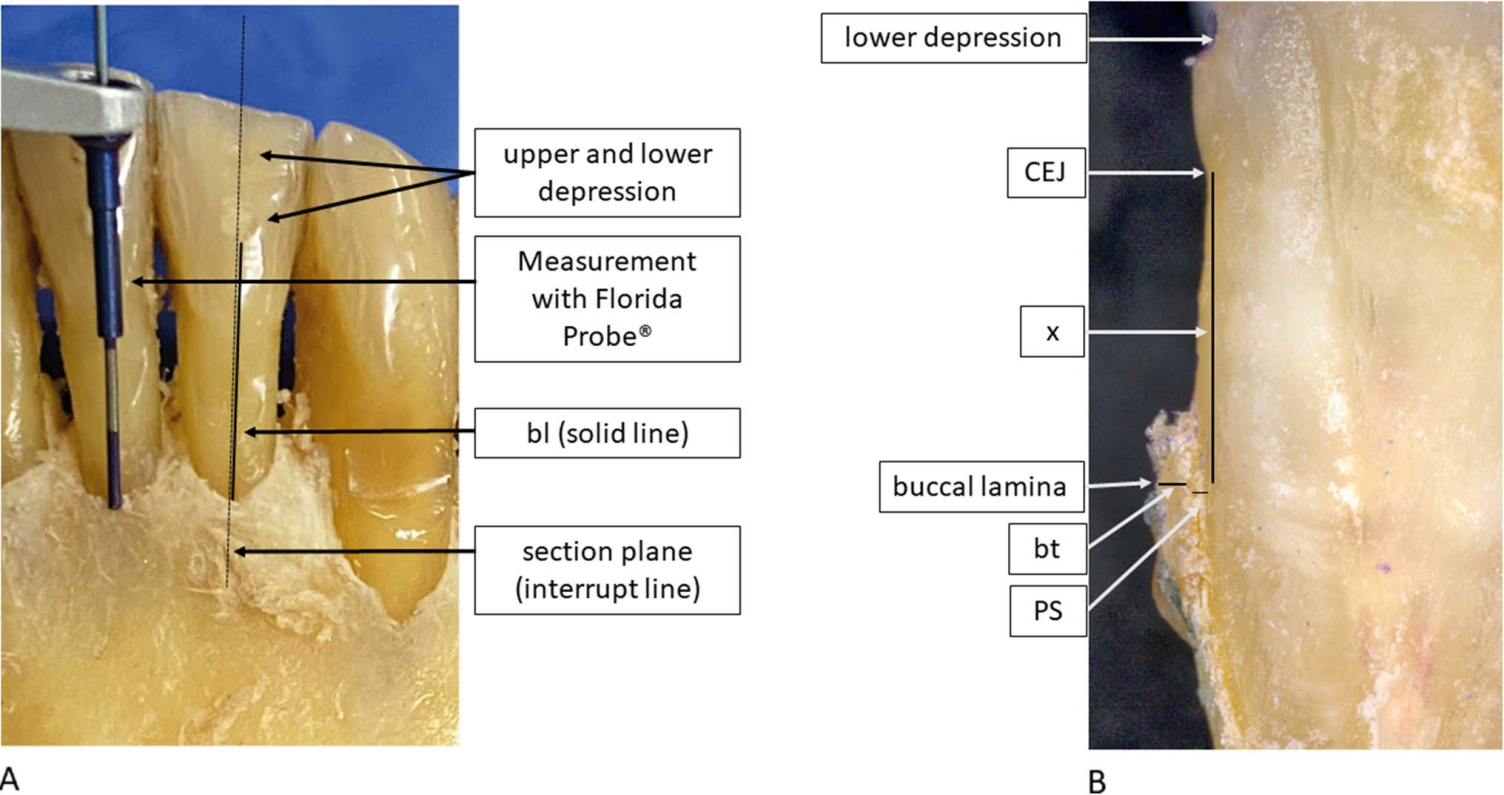
Reference measurements. **A** Clinical bl measurements. bl, buccal bone height from the most apical point of the lower depression to the most coronal point of the buccal bone. **B** Reflected-light microscopy bt measurements. CEJ, cementoenamel junction. x, random distance to a point within the first millimeter of the apical region of the buccal bone. bt, buccal bone thickness. PS, periodontal space

After radiological imaging, the gingiva was carefully removed by means of microsurgical instruments, to ensure the buccal bone was not damaged. Subsequently, in the axis of the previously milled depressions, the distance from the most apical point of the lower depression to the alveolar crest was measured on the buccal aspect of each tooth by means of a periodontal probe (Florida Probe, Clark Dental Equipment Systems Ltd, UK) with a 0.1 mm scale. Thus, a reference standard for buccal bone height (bl) measurements was established. These measurements were made by one experienced investigator (M. R.), who had previously been calibrated on a model. For calibration, the investigator had to successfully reproduce (relative agreement of 95%) the principal investigator’s (T. K.) bone-sounding measurements of clinical attachment loss at 168 sites on a standardized ex vivo reference model with a transparent gingiva (Co. M. Tech, Korea). These measurements are henceforth referred to as “probe measurements” (Fig. 2A).

Sagittal sections of the teeth and their adjacent bone were then made in the plane of the previously milled depressions. *Reflected-light micrographs* were taken of these sagittal sections at 34 × magnification (Smartzoom 5, Carl Zeiss Microscopy GmbH, Jena, Germany). A point within the first apical millimeter of the buccal bone was defined, and the buccal bone thickness (bt) was then measured at this exact location by two investigators (S. S., M. R.) in consensus. The measurements were made using the software Zen 2 core v2.5 (Carl Zeiss Microscopy GmbH). These measurements served as the reference standard for bt (Fig. 2B). To reproduce this location in CBCT, the distance from the cementoenamel junction (CEJ) to this point was noted.

### Image review

For analysis, CBCT data were exported in DICOM format into the application software OSIRIX pro (aycanOsiriX 2.06.000). Modification of contrast, magnification, orientation of the volume, and scrolling through volume in three-dimensional multiplanar reformations were allowed. All evaluations were performed on the same workstation and monitor (iMac, 27 in., Apple, California, USA) in the same dark room.

The images were reviewed by two dentists (M. R. and H. G.) with more than 8 years (M. R.) and 15 years (H. G.) of experience of CBCT diagnostics in multiplanar reconstruction. The orientation procedure was as follows: (1) the two depressions were identified, and the axis of the coronal plane was placed through the center of the depressions. (2) The axial slice was then aligned with the lower depression. (3) Measurements bl and bt were then taken in the sagittal plane.

For the measurement of bt, the investigators were given the distance to the CEJ as determined in reflected-light microscopy, to enable measurement of bt at the same location. The investigators were blinded to the reference measurements. To enable assessment of interrater and intrarater reliability, both investigators took measurements twice for each protocol, with a 2-week break between the two measurement rounds.

For calibration of the measurement protocol, the two investigators performed corresponding measurements on 40 CBCT scans of human mandibles and discussed the measurements until agreement was reached. The mandibles used for calibration were different from those that provided the 16 teeth imaged in this study.

### Statistical analysis

Because the number of teeth was restricted by the number of suitable cadaveric heads available, a sample size calculation was not performed. Measurements were descriptively documented using medians and neighboring quartiles (Q1 and Q3). Differences between clinical and radiographic measurements were calculated and described using their means and standard deviations. The Bland–Altman method for clustered data was used to create Bland–Altman plots including 95% limits of agreement. The probe measurements and reflected-light microscopy measurements were defined as reference standards. Intrarater, interrater, and intermodality agreements were assessed by means of a linear mixed model that included the jaw as a random effect and measurement 2 versus measurement 1 as a binary fixed effect. Because the number of observations per jaw was small and differed (1–6 observations per jaw), including the measurement as a random effect resulted in non-convergent models. For statistical analysis, the software R (version 4.0.5) was used in combination with the packages “lem4” and “DescTools,” for Bland–Altman plots SAS 9.4.

## Results

Three different CBCT protocols were used to image a total of 16 sites on 16 mandibular human anterior teeth. These radiographic measurements were then compared with the reference probe measurements and reflected-light microscopy measurements. One site of bl measurement was excluded because of a missing reference measurement. Consequently, bl measurements were validated on 15 teeth (Table 1).

**Table 1:** Count of teeth investigated

Tooth (FDI)	Count
33	2
32	3
31	3
41	3
42	3
43	2

*FDI*, Fédération Dentaire International (World Dental Federation)

The median distance of clinical vertical measurements (bl) was 6.4 mm (5.8–9.0). The medians of bl measurements were 6.5 mm for rater 1 (r1; 6.0–8.9) and 6.6 mm for rater 2 (r2; 6.0–9.2) for LD-CBCT-1; 6.4 mm (5.9–9.3) for r1 and 6.3 mm (5.8–9.0) for r2 for LD-CBCT-2; and 6.7 mm (5.9–9.1) for r1 and 6.7 mm (5.9–9.2) for r2 for HD-CBCT (Table 2).

**Table 2:** Medians of measurements

Device/protocol	Median bl (mm)	(Q1–Q3)	Median bt (mm)	(Q1–Q3)				
	*N* = 15		*N* = 16					
Reference standard	**6.4**	5.8–9.0	**0.5**	0.3–0.6				
	*Rater 1*				*Rater 2*			
	**Median bl (mm)**	**(Q1–Q3)**	**Median bt (mm)**	**(Q1–Q3)**	**Median bl (mm)**	**(Q1–Q3)**	**Median bt (mm)**	**(Q1–Q3)**
HD-CBCT	6.7	5.9–9.1	0.4	0.3–0.5	6.7	5.9–9.2	0.4	0.3–0.5
LD-CBCT 1	6.5	6.0–8.9	0.4	0.3–0.6	6.6	6.0–9.2	0.5	0.4–0.5
LD-CBCT 2	6.4	5.9–9.3	0.4	0.3–0.6	6.3	5.8–9.0	0.5	0.3–0.6

*bl*, buccal bone height. *bt*, buccal bone thickness. *CBCT*, cone beam computed tomography. *HD*, high dose. *LD*, low dose. Q1–Q3, neighboring quartiles

The median of bt measurements in reflected-light microscopy was 0.5 mm (0.3–0.6). In LD-CBCT-1, the median bt was 0.4 mm (0.3–0.6) for r1 and 0.5 mm (0.4–0.5) for r2; in LD-CBCT-2, it was 0.4 mm (0.3–0.6) for r1 and 0.5 mm (0.3–0.6) for r2; and in HD-CBCT, it was 0.4 mm (0.3–0.5) for r1 and 0.4 mm (0.3–0.5) for r2 (Table 2).

All linear regression coefficients were approximately 0 and showed high interrater, intrarater, and intermodality agreement. The exact values and corresponding *p*-values are listed in Tables 3, 4, and 5.

**Table 3:** Regression coefficients for interrater, intrarater, and intermodality reliability. Interrater reliability

Rater	Measurement	Protocol	Coefficient	Lower CL	Upper CL	*p*-value
1	bl	HD	− 0.017	− 0.391	0.358	0.93
	bl	LD 1	0.016	− 0.374	0.406	0.935
	bl	LD 2	− 0.002	− 0.31	0.306	0.99
2	bl	HD	0.005	− 0.34	0.349	0.979
	bl	LD 1	− 0.015	− 0.345	0.315	0.927
	bl	LD 2	0.01	− 0.318	0.338	0.952
1	bt	HD	0.009	− 0.12	0.137	0.893
	bt	LD 1	− 0.02	− 0.161	0.121	0.78
	bt	LD 2	− 0.005	− 0.131	0.121	0.938
2	bt	HD	0.001	− 0.133	0.134	0.993
	bt	LD 1	− 0.013	− 0.125	0.099	0.815
	bt	LD 2	0.003	− 0.105	0.11	0.963

**Table 4:** Regression coefficients for interrater, intrarater, and intermodality reliability. Intrarater reliability

Measurement	Protocols	Coefficient	Lower CL	Upper CL	*p*-value
bl	HD	**0.065**	− 0.293	0.423	0.719
bl	LD 1	**0.063**	− 0.295	0.422	0.727
bl	LD 2	− **0.007**	− 0.326	0.312	0.964
bt	HD	**0.002**	− 0.133	0.138	0.971
bt	LD 1	**0.003**	− 0.128	0.135	0.962
bt	LD 2	**0.009**	− 0.113	0.132	0.88

**Table 5:** Regression coefficients for interrater, intrarater, and intermodality reliability. Intermodality reliability

Measurement	Protocols	Coefficient	Lower CL	Upper CL	*p*-value
bl	LD 1 vs LD 2	− **0.046**	− 0.404	0.312	0.8
bl	LD 1 vs HD	− **0.003**	− 0.384	0.379	0.989
bl	LD 2 vs HD	− **0.049**	− 0.396	0.299	0.782
bt	LD 1 vs LD 2	− **0.008**	− 0.142	0.126	0.904
bt	LD 1 vs HD	**0.029**	− 0.108	0.166	0.671
bt	LD 2 vs HD	**0.021**	− 0.109	0.152	0.747

*bl*, buccal bone height. *bt*, buccal bone thickness. *CL*, confidence level. *df*, degrees of freedom. *HD*, high-dose cone beam computed tomography. *LD*, low-dose cone beam computed tomography

**Fig. 3:**
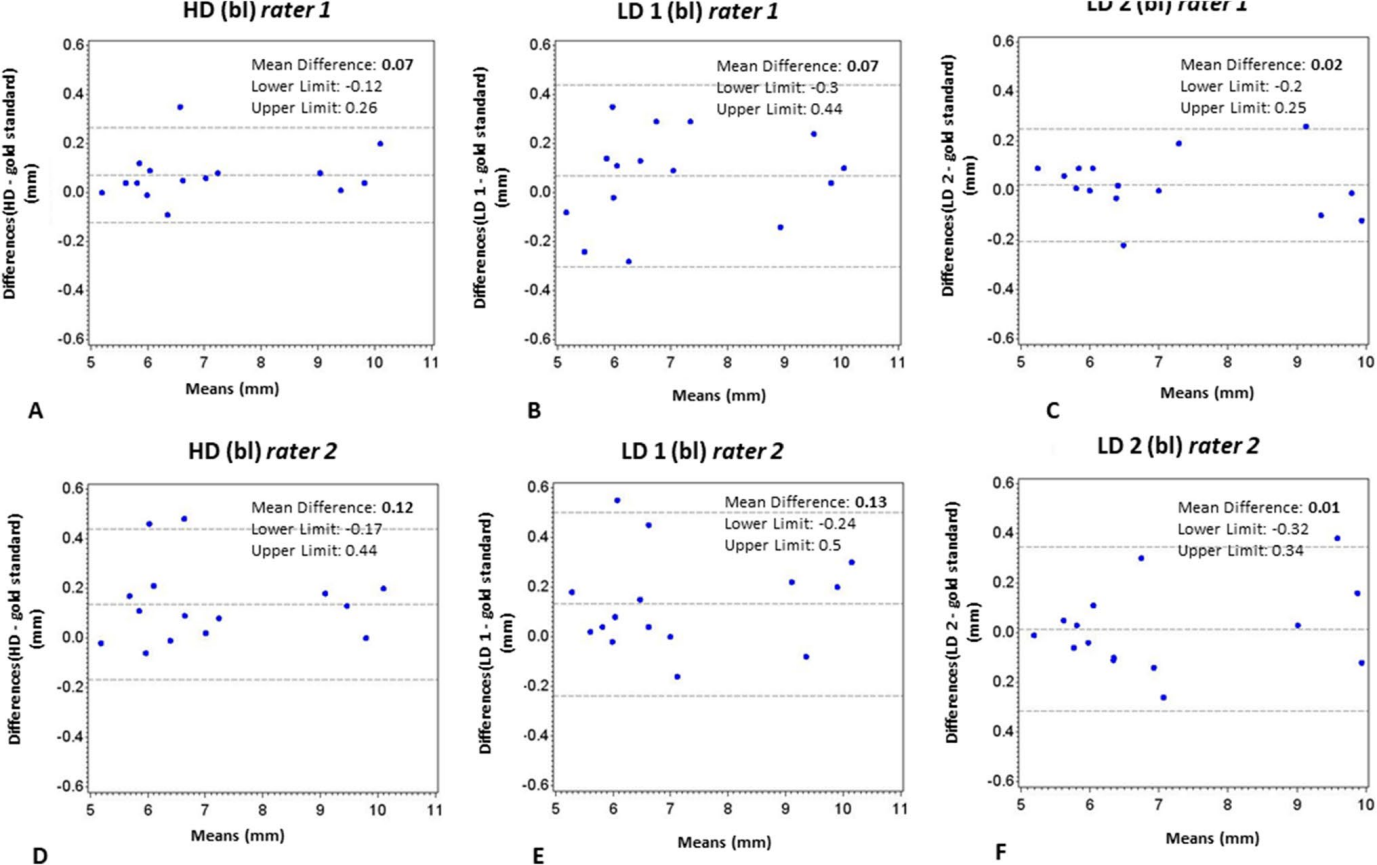
Bland–Altman plots of bl measurements. **A**–**C** Bland–Altman plots of bl of different CBCT protocols (HD, LD1, LD2) compared with reference standard (probe measurements) of rater 1. **D**–**F** Bland–Altman plots of bl of different CBCT protocols (HD, LD1, LD2) compared with reference standard (probe measurements) of rater 2. bl, buccal bone height. CBCT, cone beam computed tomography. HD, high dose. LD, low dose

**Fig. 4:**
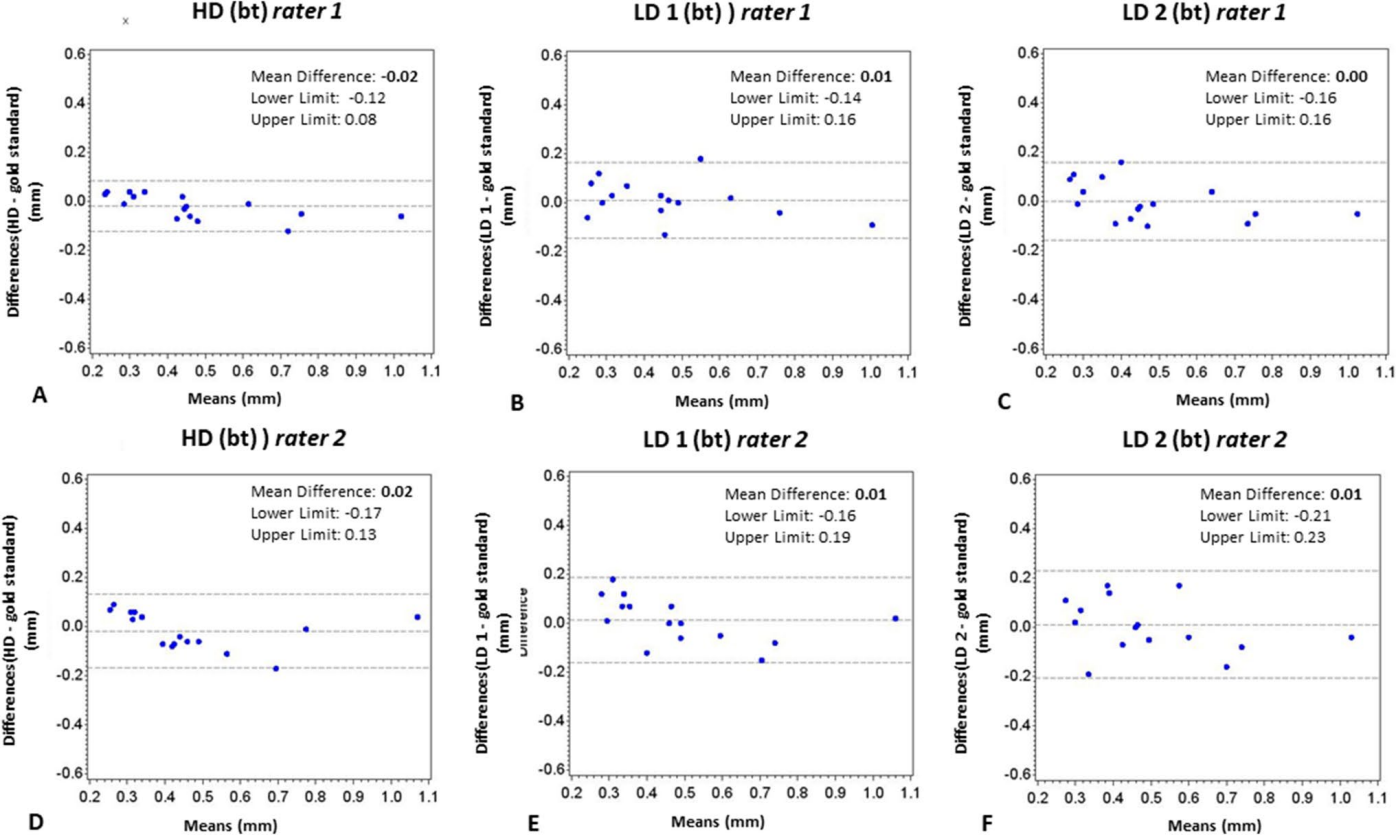
**Bland–Altman plots of bt measurements**. **A–C** Bland–Altman plots of bt of different CBCT protocols (HD, LD1, LD2) compared with reference standard (reflected-light microscopy) of rater 1. **D–F** Bland–Altman plots of bt of different CBCT protocols (HD, LD1, LD2) compared with reference standard (reflected-light microscopy) of rater 2. bt, buccal bone thickness. CBCT, cone beam computed tomography. HD, high dose. LD, low dose

Bland–Altman analysis revealed mean differences of bl measurements compared with reference measurements of 0.07 mm (r1) and 0.12 mm (r2) for HD-CBCT; 0.07 mm (r1) and 0.13 mm (r2) for LD-CBCT-1; and 0.02 mm (r1) and 0.01 mm (r2) for LD-CBCT-2. For bt measurements, the mean differences were 0.02 mm (r1) and 0.02 mm (r2) for HD-CBCT; 0.01 mm (r1) and 0.01 mm (r2) for LD-CBCT-1; and 0.00 mm (r1) and 0.01 mm (r2) for LD-CBCT-2. The 95% limits of agreement can be seen in Figs. 3 and 4.

## Discussion

The hypothesis that LD-CBCT is suitable for imaging buccal bone adjacent to mandibular anterior teeth in this specific experimental setting was confirmed for the two CBCT devices used.

All protocols showed high agreement with the reference probe and reflected-light microscopy measurements for both raters. The analysis indicated very similar medians and neighboring quartiles for the bt and bl measurements in all CBCT protocols. The HD protocol did not seem to be superior in this regard.

The 95% limits of agreement were also similar among all CBCT protocols with respect to bt irrespective of the investigator. Maximum deviations ranged from − 0.20 to 0.22 mm. Mean values were all close to 0. There was no systematic over- or underestimation of buccal bone thickness in any of the CBCT protocols. Similar results were also seen with respect to bl. All protocols revealed similar difference in means. The 95% limits of agreement ranged between − 0.3 and 0.50. These differences were all below 1 mm and therefore within the range of periodontal probe measurement errors [21, 22]. A closer look reveals that the range of deviations was slightly smaller in HD-CBCT. This may reflect the better subjective image quality of HD-CBCT, as the result of using a higher radiation dose. The results are consistent with a study performed on pig jaws and with studies involving older generations of devices [14, 23].

Linear regression coefficients indicated high interrater and intrarater reliability for all protocols. Confidence levels were highest between − 0.40 mm and 0.42 mm for bl and − 0.16 mm and 0.16 mm for bt. These values are within an acceptable range from a clinical perspective. *p*-Values were not significant, but this absence of significance must be interpreted with caution because of the relatively small sample size. A strength of this study is that reflected-light microscopy provides a very high-quality reference standard [24]. It was therefore possible to clearly determine whether bone was present and how thick it was. This strengthens the significance of the results of this study.

Another strength of the study was the presence of soft and hard tissues (cervical vertebrae with adjacent muscles, tongue, base of skull, etc.), whose absence in older studies — thus leading to unreliable “better” results — often had to be regarded as a limitation.

The DAPs of the two LD-CBCT protocols (69 mG ycm^2^ [protocol 2; Orthophos 3D SL] and 87 mGy cm^2^ [protocol 1; Veraview X800]) were significantly lower than the DAP of the HD-CBCT protocol (1396.95 mGy cm^2^; Veraview X800). Dentsply Sirona, the manufacturer of the device used for the LD-CBCT-2 protocol, gives a DAP of 943 mGy cm^2^ for HD-CBCT protocols [13]. Thus, the DAPs of the two LD protocols used correspond to 6% (Veraview X800) and 7% (Orthophos 3D SL) of the DAPs of the corresponding HD protocols of the same CBCT devices. As an example, digital panoramic X-rays can have a DAP of 28 mGy cm^2^, whereas the DAP of analog panoramic views is approximately 88 mGy cm^2^, which is higher than the DAP of the LD protocols used in the present study [25]. In addition, panoramic views can only represent two dimensions. A full mouth status using digital technology has a DAP of approximately 67 mGy cm^2^ and is thus in a similar range to the DAPs of digital and analog panoramic views; however, it also only represents the structures in two dimensions [25]. In general, this raises the question of whether LD-CBCT has the potential to replace panoramic views in periodontology in the long term. Reviews have proven the benefits of CBCT in periodontology [26, 27]. Studies using older devices showed that, in an *ex vivo* setting similar to that in the present study, horizontal bone resorption can be more reliably visualized with CBCT than with dental films [17]. Studies of the visualization of furcation defects also showed very good results with an HD protocol [28]. Therefore, the suitability of LD protocols for imaging different periodontal structures and clinical issues should be investigated further in future studies. As mentioned earlier, LD-CBCT might also be an alternative imaging technique for orthodontists. The DAP of a lateral cephalometric image acquired with one of the devices used in this study (the Orthophos 3D SL) ranges from 22 to 26 mGy cm^2^ [29]. When combined with panoramic views, as is often the case in orthodontics, this generates a DAP similar to that of LD-CBCT.

As described in the introduction, nonionizing imaging modalities such as ultrasound can be used to image buccal bone, but they are not as ubiquitous as LD-CBCT devices are likely to be in future. At least in Germany and Switzerland, conventional CBCT devices are already widely available. If manufacturers later offer their devices with additional LD protocols, it can be assumed that these will also be available for many patients [19, 30].

In the present study, buccal bone thickness ranged between 0.22 and 1.05 mm. The results showed high agreement for LD-CBCT at all 16 sites. These results are superior to those in earlier studies investigating conventional CBCT protocols, which showed that bone visualization is harder in CBCT once the bone has fallen below a certain thickness, for example, 0.78 mm around implants [31]. The superiority of the present results might be because a newer generation of device was used, which includes a newer generation of sensors, a smaller voxel size, and a reduction of the partial volume effect [32, 33].

### Limitations

The sample size of 16 teeth was low. The number of teeth was limited by the number of human donors available and their dental status. Nonetheless, the high levels of agreement with the reference standards and between the two investigators for all protocols indicate that the results are highly reliable.

The *ex vivo* nature of the experiment meant there was no risk of natural motion, such as tremors, which can lead to motion artifacts. These artifacts can significantly reduce the quality and information content of the image. Even the human heartbeat has been discussed as a cause of motion artifacts [33–35]. None of the teeth investigated had metallic restorations, which can also cause imaging artifacts that can potentially affect visualization of the buccal bone [36].

In our study, we only used half heads. To mimic the missing half, gel pads were used as described in “Materials and methods”. However, these pads cannot imitate bony structures or even the teeth and restorative materials and the artifacts caused by these structures. Thus, image quality for a half head may be slightly better than it is for a complete head [20, 36].

Another limitation of this experimental setting is the fact that measurements were not fully automated and windowing was allowed. This may have led to voxel interpolation by the software. After software manipulation, the human eye “locates” the margin of the bone irrespective of the voxel sizes. The application of different filter settings can cause differences in measurements between two raters and even within one rater [33]. The radiographic measurements in this study were performed by two experts, because the main objective was to test and compare the performance of the protocols. However, it has already been shown — e.g., in the field of endodontics — that diagnostic results in CBCT findings are highly dependent on examiner experience [37]. Similar effects must be assumed for the present study. An existing study on the reliability of measurements of marginal bl showed that interrater reliability is lower when examiners’ differing levels of experience are taken into account [38].

## Conclusion

Overall, the results of this study are promising regarding the future use of LD-CBCT. Within the limitations of the study, they show that LD-CBCT is a highly accurate and reliable method for detecting and measuring the alveolar buccal bone and its thickness adjacent to mandibular anterior teeth. Future studies should investigate the extent to which other periodontal indications can be assessed with LD-CBCT and confirm this ex vivo result in a clinical setting.

## Supplementary Information

Below is the link to the electronic supplementary material.

Supplementary file1 (ODS 5 KB)

Supplementary file2 (PDF 101 KB)
