# Supplementary material for: Low-dose CBCT imaging of alveolar buccal bone adjacent to mandibular anterior teeth— a pilot study
Source: Clin Oral Investig. 2022 Feb 1;26(5):4173–82. doi: 10.1007/s00784-022-04389-x (PMC9072474; doi:10.1007/s00784-022-04389-x)
Supplement: Supplementary file 2 — Supplementary file2 (PDF 101 KB) [file 784_2022_4389_MOESM2_ESM.pdf]

Certification of English Editing

To whom it may concern

An experienced editor whose first language is English has carefully reviewed this manuscript entitled *Low-dose CBCT imaging of alveolar buccal bone adjacent to mandibular anterior teeth—A pilot study.*

I specialize in editing papers written by physicians and scientists whose native language is not English.

Please address *specific* criticism of the use of English in this manuscript to the email address given below.

Signature

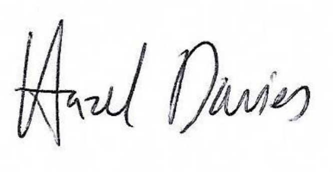

Hazel Davies MSc (UCL)
Ely, UK
17/12/2021

Email: hazel@hazellanguageservices.co.uk

Member of the *European Association of Science Editors*
